# Developing an active lifestyle for children considering the Saudi vision 2030: The family’s point of view

**DOI:** 10.1371/journal.pone.0275109

**Published:** 2022-09-26

**Authors:** Ahmed Hassan Rakha, Dekheel Mohamed Albahadel, Hany Abdelaziz Saleh

**Affiliations:** 1 Department of Physical Education and Kinesiology, College of Education, Qassim University, Buraidah, Saudi Arabia; 2 Department of Curriculum and Teaching Methods of Physical Education, Faculty of Physical Education, Port-Said University, Port Fouad City, Egypt; 3 Department of Psychology, College of Education, Qassim University, Buraidah, Saudi Arabia; 4 Department of Sports Training and Movement Science, Faculty of Physical Education, Port-Said University, Port Fouad City, Egypt; Universiti Malaya, MALAYSIA

## Abstract

Motivating children to lead a healthy and active lifestyle is a family mission and a responsibility shared by society. This study is important in developing the family’s role in promoting children’s active lifestyle. This study aims to develop the role of the family in promoting an active lifestyle for children in light of the Saudi vision 2030. The sample included 405 parents who filled out an online survey about their children aged 3–12 years. The results show families’ lack of interest in applying the WHO standards and the guidelines of the Saudi Ministry of Health regarding children’s regular daily physical activity. The families are reluctant to involve children in sports clubs. The parents desire to employ crews that enhance the movement and recreational activities of children in shopping centers and parks. The results also show that the presence of the father and mother together inside the house and their educational level have an impact on the effectiveness of the positive reinforcement of the active lifestyle of the children. To promote an active lifestyle for children, there is a need to determine why some families are hesitant to enroll their children in sports clubs. Additionally, there is a need to develop media and awareness campaigns for families in order to achieve the desired goals of developing an active lifestyle for children, which are approved by the WHO and Saudi Ministry of Health standards and are consistent with families’ low educational levels.

## Introduction

Sedentary behavior and physical inactivity increase the risk of many chronic diseases and shorten life expectancy [1]. The prevalence of obesity among children in recent decades has increased dramatically [2]. Treatment of obesity is difficult, and overweight children are more likely to become obese adults. These trends have led member states of the World Health Organization (WHO) to target no increase in childhood obesity by 2025. According to Farooq et al. [3], moderate-intensity physical activity is important for preventing and treating childhood obesity, but its effects decline with age. Therefore, childhood should be the time to promote moderate-intensity physical activity [4].

Childhood divided into three stages: early childhood (3–5 years old), middle childhood (6–8 years old), and late childhood (9–11 years old). The stages reflect the child’s cognitive, physical, psychological, and social development [5]. Early childhood as a period of rapid physical and cognitive development, characterized by the formation of habits and adaptations to lifestyles and the WHO has developed guidelines for physical activity, sedentary behavior, and sleep for children under 5 years old [6]. The Saudi Ministry of Health, through its guideline issued within the Agility Program, clarified the basic standards for an active lifestyle for the age group 5–17 years; these standards included periods of physical activity, sedentary activities, and the number of hours of sleep, and appropriate nutrition [7].

Motivating children to lead a healthy and active lifestyle is a family mission and a responsibility shared by the family and society [8–12]. Many studies have addressed the family’s roles in promoting the active lifestyle of their children and how best to develop and improve children’s lives [4, 10, 13–15].

In April 2016, Saudi Arabia launched Vision 2030, which included three main axes: a vibrant society, a thriving economy, and an ambitious nation. Vision 2030 established 96 strategic goals and 13 programs to achieve those goals. A vibrant society axis aims to create a productive and strong society by enabling families to fulfill their roles and responsibilities in establishing the best social and health care system possible. The vision also paid great attention to the importance of practicing physical activity, developing the individual’s health, and improving the individual’s lifestyle [16, 17].

Despite the efforts made to promote a healthy lifestyle in all regions of Saudi Arabia as part of Vision 2030, the percentage of individuals participating in physical activities remains low. According to a study conducted by The General Authority for Statistics KSA [18] the percentage of individuals practicing sports activity for 150 min or more each week was just 17.40% of the total population aged 15 years and over.

In addition, the rates of obesity and inactivity continue to be high. According to the Saudi Ministry of Health statistics for 2020, the obesity rates among Saudis reached 59%; 28.7% of them exceeded the term overweight and became obese, while 31% were overweight in the age group of 15 years and above. The percentage of obesity among children enrolled in pre-school reached 6%, while it reached 9.3% among school-age children. This indicates that 60% of Saudis do not sufficiently engage in physical activities [19]. Elkhodary and Farsi [20] also studied the relationship between obesity, physical activity, social and economic factors, and lifestyle among Saudi children and adolescents, identifying physical inactivity and time spent watching television as important risk factors for obesity among adolescents and Saudi schoolchildren.

Here we investigate an active lifestyle among children in Qassim Province and the role of the family in promoting an active lifestyle for children in light of the vision of the Kingdom of Saudi Arabia 2030.

## Materials and methods

### Ethics statement

A completely voluntary and anonymous online survey was conducted in the present study. Additionally, no compensation was provided. The informed consent was obtained from participants via an online form in which the first question was "Do you wish to participate?" When the respondent answered "yes," the axis moved forward. If the response was refused, the questionnaire was closed with a thank you. We also ensured that the survey presented no material/question that might have caused any negative impact or harm to children. Moreover, participants were informed that they could withdraw at any time without giving any reason or facing any negative consequences. There was no way to obtain additional informed consent from parents on behalf of the children (3–12 years old). Due to the survey’s anonymous nature, the survey could not have obtained such informed consent from parents. The study was conducted according to ethical approval obtained as a written certificate from the Regional Research Ethics Committee at the Ministry of Health–Qassim Province, which registered at the national committee of bioethics (NCBE) at King Abdulaziz City for Science and Technology, KSA (APPROVAL NUMBER/ 485360–1443).

### Study questions

In order to accomplish the purpose of this study, the following questions were formulated:

1. What role does the family play in encouraging their children to live an active lifestyle?
2. What is the level of parental awareness of community resources needed to promote active lifestyles in children?
3. How effective are community activities and awareness programs for family roles in promoting a child’s active lifestyle?
4. Are there statistically significant differences in the sample responses about the necessity of developing an active lifestyle for children due to differences (Marital Status, Parent’s Education Level) at the level (α ≤ 0.05)?

### Design

A descriptive cross-sectional study was adopted using an online questionnaire that included closed and open questions to explore the realities of the children’s active lifestyle through their parents’ responses.

### Study population

The study population included families who live in Qassim province and have children ranging in age from 3 to 12 years. To determine the approximate number of children in Qassim province, the (Noor) database system of the General Education Department was used. The number of children enrolled in kindergarten and primary school was 105,845 for the academic year 2020/2021 [21]. We use Steven Thompson’s formula to calculate the sample size from the next formula

$$n=\frac{N\times p(1-p)}{\left[[N-1\times (d^{2}÷z^{2})]+p(1-p)\right]}$$

where (N: Population size = 105.845, z:Confidence level at 95% = 1.96, d:Error Proportion = 0.05, p: Probability = 50) [22]. The sample size required to represent the population was determined to be *n* = 383 children.

### Study sample

The study sample consisted of 405 parents who filled out an online survey about their children aged 3–12 years old in the Qassim province. S1 Table shows a description of the study sample.

### Questionnaire

#### Designing the questionnaire’s axes and their items

Based on several related studies [4, 10, 13, 15], the questionnaire initially included the following axes and their items in order to answer the study questions: Axis 1) Demographic information (10 items). Axis 2) The family has a significant role in promoting an active lifestyle (i.e., exercise, physical and sports activities) among their children (15 items). Axis 3) Parental awareness of community resources needed to promote active lifestyles in children (12 items). Axis 4) The effectiveness of community activities and awareness programs to promote children’s active lifestyle (7 items).

The first eight items on the second axis were written as multiple-choice questions depending on their goals, and they were ranked from best to worst based on active, healthy lifestyle best practices. The rest of the items were written on a 5-point Likert scale, as shown in S2 Table which was used to determine the sample’s acceptability level [23].

#### Validity and reliability of the questionnaire

*Content validity*. Five experts in sports sciences, physical activity, sociology, and childhood reviewed the initial questionnaire. They revised it and confirmed the validity of the scientific content of the items and their association with the axis in terms of content. The final version, divided into three axes, included 34 items, other than the first axis, which aimed to collect demographic information via 10 items [24].

*Internal consistency validity*. Due to the COVID-19 pandemic and the related preventive measures, we designed an online version of the final questionnaire using a Google Form. We published the link through social media applications (Facebook, Snapchat, Instagram, Twitter, and WhatsApp) until 30 responses were collected in the period from 6 to 20 December 2020. This data was used as a pilot sample to confirm the questionnaire’s internal consistency, validity, and reliability.

S3 Table shows the correlation (*R*) and significance level (*p*) between every item and their axis. Given that the items on axis 2 are not scaled the same, the raw scores were converted into standardized (*Z*) scores [25]. The Pearson correlation coefficient was calculated between each item and the mean degree of axis 2: *r*(28) = 0.41:0.71, p<0.01. For axis 3, *r*(28) = 0.47:0.82 *p*<0.01, while or axis 4, *r*(28) = 0.56:0.91 *p*<0.01. These are ranged from a moderate to a strong positive correlation. Accordingly, the internal consistency validity is confirmed between items and their axis [26].

*Reliability*. For axis 2, Cronbach’s alpha is based on standardized items and is used because the items are not scaled similarly [27]. We use Cronbach’s alpha to determine the questionnaire reliability for others axes because they used a 5-point Likert scale.

The Cronbach’s alpha value based on standardized items for axis 2 is 0.81 (S4 Table). In addition, the Cronbach’s alpha values for axes 3 and 4 are 0.90 and 0.82, respectively. These are greater than 0.7 and therefore indicate good reliability [28].

### Basic study

The study was conducted from 20 January 2020 to 22 February 2021. The questionnaire’s link was published through social media (e.g., Facebook, Snapchat, Instagram, Twitter, and WhatsApp). The sample should include only families in the Qassim province with children aged 3–12. Any responses that did not fit the sample criteria were excluded.

### Statistical analysis

IBM SPSS Statistics for Windows (version 25, IBM Corp, Armonk, NY, USA) was used for the following statistical analyses: Frequencies, Percentage (*%*), Mean (*M*), Standard deviation (*SD*), Cronbach’s alpha (α), Kolmogorov-Smirnov normality test, Kruskal-Wallis test (*H*), and Mann-Whitney test (*U*).

## Results

### Research question 1, “What role does the family play in encouraging their children to live an active lifestyle?”

Table 1 shows that parents were not interested in registering children in various sports federations, as the number of children not registered in sports federations reached 92.6%. The children practiced sports randomly. Most children practice sports for less than an hour, two to three times a week. The random unregulated sports practice percentage for children was about 60%. On average, 36% of children practiced sports for less than one hour, and 22% practiced two to three times per week.

**Table 1:** Descriptive statistics of the family’s role in enhancing the child’s physical exercise and sports activity.

Statements	Response	*n* *%*	*M* *SD* Result
1- Is the Child registered in one of the sports federations?	Yes	30 7.4	1.07 0.26 No
	No	375 92.6	
2- Does your Child engage in sports or any physical activity?	Yes, regularly under the supervision of a specialist coach	34 8.4	1.77 0.59 Yes, irregularly (random—not purposeful)
	Yes, irregularly (random—not purposeful)	242 59.8	
	Do not exercise	129 31.9	
3- How often does the child exercise sports and physical activity per week?	Daily	90 22.2	2.97 1.48 2–3 times
	More than three times	67 16.5	
	2–3 times	88 21.7	
	One time	61 15.1	
	Do not exercise	99 24.4	
4- How long do the child exercise sports and physical activity per day?	60 minutes (an hour)—regular sports activity (the child is regular in training under the supervision of a coach)	44 10.9	2.52 1.22 Less than an hour—general physical activity (includes free play, walking, running, etc.)
	180 s (2.5 hours)—general physical activity (includes free play, walking, running, etc.)	34 8.4	
	1–2 hours—general physical activity (includes free play, walking, running… etc.)	96 23.7	
	Less than an hour—general physical activity (includes free play, walking, running, etc.)	145 35.8	
	Do not exercise	86 21.2	
5- Where the Child engages in sports and physical activity?	Official government sports club	16 4.0	2.80 1.42 Outside the house with his peers
	District Sports Club	15 3.7	
	Private gym	40 9.9	
	Parks and gardens	75 18.5	
	Outside the house with his peers	216 53.3	
	At Home	26 6.4	
	Do not exercise	17 4.2	
6- Is there kinetic toys and sports equipment suitable for the Child at home?	Yes	256 63.2	1.63 0.48 Yes
	No	149 36.8	
7- Is there sports equipment suitable for parents at home?	Yes	186 45.9	1.46 .50 No
	No	219 54.1	
8- Are parents keen on exercise, sports, and physical activity?	Yes, regularly	42 10.4	1.89 0.56 Yes, irregularly
	Yes, irregularly	275 67.9	
	Do not exercise	88 21.7	
9- Does the family encourage the child to practice sports at home?`	Strongly Agree	115 28.4	3.48 0.99 High
	Agree	156 38.5	
	Neutrally	99 24.4	
	Disagree	25 6.2	
	Strongly Disagree	10 2.5	
10- Does the family set a specific time for the child to practice sedentary activities (watching TV—PlayStation—Smartphones, etc.)?	Strongly Agree	117 28.9	3.70 1.15 High(3)
	Agree	132 32.6	
	Neutrally	96 23.7	
	Disagree	37 9.1	
	Strongly Disagree	23 5.7	
11- Is the family aware of sufficient information about the healthy food system for children?	Strongly Agree	84 20.7	3.60 1.07 High
	Agree	154 38.0	
	Neutrally	110 27.2	
	Disagree	36 8.9	
	Strongly Disagree	84 20.7	
12- Does the family ensure that the child follows a healthy diet?	Strongly Agree	81 20.0	3.46 1.12 High
	Agree	119 29.4	
	Neutrally	131 32.3	
	Disagree	48 11.9	
	Strongly Disagree	24 5.9	
13- Is The family aware of the necessary information about the child’s healthy sleep habits?	Strongly Agree	93 23.0	3.72 1.02 High
	Agree	169 41.7	
	Neutrally	96 23.7	
	Disagree	31 7.7	
	Strongly Disagree	16 4.0	
14- Is the family keen to follow the child’s healthy sleep habits (sleep early—sleep enough)?	Strongly Agree	101 24.9	3.75 1.03 High (2)
	Agree	157 38.8	
	Neutrally	105 25.9	
	Disagree	23 5.7	
	Strongly Disagree	17 4.2	
15- Do you think that parents’ social and psychological stability enhances the children’s active life?	Strongly Agree	238 58.8	4.45 0.77 High (1)
	Agree	124 30.6	
	Neutrally	37 9.1	
	Disagree	1 0.2	
	Strongly Disagree	5 1.2	

In addition, 53% of children engaged in sports and physical activity outside the house with their peers, 4% of children practiced sports in government clubs, and 3.7% were members of a district sports club. The results also indicated that 63.2% of families had kinetic toys and sports equipment suitable for the child at home, while among 55% of respondents, suitable sports equipment was available at home for the parents. The results showed that 68% of fathers practiced sports and physical activity, but irregularly.

The average responses to items 9 to 15, which used a five-point Likert scale to determine parents’ attitudes toward the role of the family in promoting active lifestyles in children, were high, ranging from strongly agree to agree. Question 15 obtained the top degree of agreement (*M* = 4.45, *SD* = 077), and question 12 obtained the lowest degree of agreement (*M* = 3.46, *SD* = 1.12). Therefore, the results indicate that the parents encouraged children to practice sports activities, set a specific time for sedentary activities during the day, be acquainted with healthy diets, and acquire healthy sleep habits. Moreover, they realized the importance of social and psychological stability between parents and its essential role in promoting an active lifestyle in a child.

### Research question 2, “What is the level of parental awareness of community resources needed to promote active lifestyles in children?”

The results of the axis of parental awareness of community resources needed to promote active lifestyles in children are shown in Table 2. The average response of parents to items 2, 8, and 9 was high, ranging from strongly agree to agree. Concerning item 8, which concerned the school’s effectiveness in encouraging and rehabilitating children through physical education and sports activities, it placed first (*M* = 3.44, *SD* = 1.18). It was followed by item 2, which referred to parents’ agreement with the statement that neighborhood clubs provided children with the opportunity to participate in various sports activities (M = 3.43, SD = 1.23). Finally, item 9 referred to parental consent in their response to the availability of a public park in each residential neighborhood that enabled the family and children to engage in physical activity (*M* = 3.42, *SD* = 1.26).

**Table 2:** Descriptive statistics on parental awareness of community resources needed to promote active lifestyles in children.

*Statements*	*Strongly Agree%*	*Agree %*	*Neutrally %*	*Disagree %*	*Strongly*	*M*	*Rank*	*Level*
					*Disagree %*	*SD*		
*1- Governmental sports clubs are influential and provide children with opportunities to practice various sports activities*	96	91	111	60	47	3.32	5	Neutral
	23.7	22.5	27.4	14.8	11.6	1.30		
*2- The District Sports Club project is influential and provides children with opportunities to practice various sports activities*	96	109	108	58	34	3.43	2	High
	23.7	26.9	26.7	14.3	8.4	1.23		
*3- Fees for the child’s participation in government sports clubs appropriate to the family’s income level*.	54	84	162	65	40	3.12	7	Neutral
	13.3	20.7	40.0	16.0	9.9	1.13		
*4- Fees for the child’s participation in the District Sports Clubs appropriate to the family’s income level*.	57	105	167	47	27	3.30	6	Neutral
	14.1	25.9	41.2	11.6	6.7	1.07		
*5- Fees for the child’s participation in private sports clubs and academies commensurate with the family’s income level*.	36	87	134	88	60	2.88	11	Neutral
	8.9	21.5	33.1	21.7	14.8	1.17		
*6- Government sports clubs have specialized qualified trainers to train children in various sports activities (football—basketball—volleyball—handball—swimming—field and track competitions—gymnastics—etc*.*)*	49	93	132	79	52	3.02	9	Neutral
	12.1	23.0	32.6	19.5	12.8	1.19		
*7- The District Sports Clubs have specialized qualified trainers to train children in various sports activities (football—basketball—volleyball—handball—swimming—field and track competitions—gymnastics*.*)*	49	64	131	102	57	2.87	12	Neutral
	12.1	15.8	32.3	25.2	14.1	1.20		
*8- The school encourages children to exercise and physical activity and rehabilitating them physically and healthily through physical education and sports activities*.	89	112	125	47	32	3.44	1	High
	22.0	27.7	30.9	11.6	7.9	1.18		
*9- In every residential neighborhood*, *a public park enables the family to practice physical activity*.	102	103	98	67	35	3.42	3	High
	25.2	25.4	24.2	16.5	8.6	1.26		
*10- In Public Parks*, *There is sports equipment suitable for children to practice physical activity*.	75	99	156	55	20	3.38	4	Neutral
	18.5	24.4	38.5	13.6	4.9	1.08		
*11- In public parks*, *Guide instructional panels are available for the use of sports equipment*, *and there are safety and security factors for children to practice physical activity*.	63	77	133	97	35	3.09	8	Neutral
	15.6	19.0	32.8	24.0	8.6	1.18		
*12*. *Private sports clubs and academies are available enough for children to exercise in various sports activities*, *and it is near the family home*.	58	76	119	97	55	2.96	10	Neutral
	14.3	18.8	29.4	24.0	13.6	1.24		

On the other hand, the parent’s responses to the rest of the items were neutral, i.e., between agree and not agree. This indicates that the family was not aware of the opportunities for exercise and physical activity that government clubs provided to children, nor of the fee for participating in them; additionally, they were not aware of a neighborhood or private clubs and the level of qualified trainers working in them. Furthermore, they were unaware of the numerous private sports centers where children could participate in various sports activities, the availability of sports equipment and tools in public parks suitable for children, and instructions on how to use them. The average response of the parents to these items ranged from (*M* = 2.87, *SD* = 1.20) and (*M* = 3.38, *SD* = 1.08).

### Research question 3, “How effective are community activities and awareness programs for family roles in promoting a child’s active lifestyle?”

Table 3 shows the results of the axis of the quality of community activities and awareness programs in promoting an active lifestyle. It reveals that parents’ average responses to items 2, 3, and 5 were high, with item 2 coming in first, followed by 3, indicating parents’ desire to hire animation crews in public parks and marketing centers to organize sports programs, workshops, and creative activities for visitors to enhance their children’s active lifestyle. The means were (*M* = 3.97, *SD* = 1.05) and (*M* = 3.88, *SD* = 1.12). Item 5 came in third place, indicating that parents are interested in following up with awareness programs through various media to acquire the necessary information about children’s active lifestyles (*M* = 3.43, *SD* = 1.04).

**Table 3:** Descriptive statistics of the quality of community activities and awareness programs to promote an active lifestyle.

Statements	*Strongly Agree%*	*Agree %*	*Neutrally %*	*Disagree %*	*Strongly Disagree %*	*M SD*	*Rank*	*Level*
1- Does community activities such as (Dates Festival—Buraidah spring, etc.) organized by responsible government agencies allow the child to participate in physical activities and sports competitions that promote an active lifestyle?	69	95	138	74	29	3.25	6	Neutral
	17.0	23.5	34.1	18.3	7.2	1.15		
2—It is necessary to employ an animation crew in public parks to organize sports programs, workshops, and creative activities for visitors to enhance the active lifestyle of children	151	142	71	29	12	3.97	1	High
	37.3	35.1	17.5	7.2	3.0	1.05		
3- It is necessary to employ an animation crew in Shopping malls to organize sports programs and activities for visitors to enhance the active lifestyle of children.	146	134	69	41	15	3.88	2	High
	36.0	33.1	17.0	10.1	3.7	1.12		
4- The specialized organizations (Sports Ministry—Health Ministry, etc.) provide the necessary awareness programs to help the family in promoting an active lifestyle for children	63	87	148	63	44	3.15	7	Neutral
	15.6	21.5	36.5	15.6	10.9	1.19		
5- Are you keen to follow up awareness programs through various media to develop the necessary information about children’s active lifestyles?	71	115	155	46	18	3.43	3	High
	17.5	28.4	38.3	11.4	4.4	1.04		
6- Is the family has sufficient information to meet the World Health Organization and the Saudi Ministry of Health standards regarding the child’s practice of physical activity?	65	137	128	41	34	3.39	4	Neutral
	16.0	33.8	31.6	10.1	8.4	1.13		
7- Is the family committed to achieving the World Health Organization and the Saudi Ministry of Health standards regarding the child’s practice of physical activity?	67	128	122	54	32	3.36	5	Neutral
	16.5	31.6	30.1	13.3	7.9	1.14		

The parent’s responses to the remaining items were neutral, indicating their lack of awareness of community activities organized by responsible government agencies that allow the child to participate in physical activities and sports competitions that promote an active lifestyle, as well as the awareness programs organized by the Ministry of Health and Sports to develop information about an active lifestyle. Furthermore, there was a lack of parental awareness of WHO and Saudi Ministry of Health guidelines for children’s physical activity. The average response of the parents to these items ranged from (*M* = 3.15, *SD* = 1.19) and (*M* = 3.39, *SD* = 1.13).

### Research question 4, “Are there statistically significant differences in the sample responses about the necessity of developing an active lifestyle for children due to differences (Marital Status, Parent’s education level) at the level (α ≤ 0.05)?”

#### The normality test

*H*_0_: The data follows a normal distribution.

S5 Table shows the Kolmogorov-Smirnov normality test results showed that sample’s average responses on the questionnaire’s axes did not reflect the normal distribution of the three axes according to the Kolmogorov-Smirnov test results. *D* (405) = 0.53, *P* = 0.01; *D* (405) = 0.71, *p≤0*.*01*; *D* (405) = 0.66, *p≤0*.*01*, respectively. Thus, we reject the null hypothesis and use the nonparametric tests to determine if there are significant differences in the mean of the sample’s responses according to parent’s marital status and education level.

#### The significance of the differences in the mean of the sample’s responses according to parent’s marital status

*H*_1_: There are statistically significant differences between the mean responses of the sample on axes 2 and 3 based on the Marital Status of the Parent at the 0.05 significance level.

From Table 4, The Kruskal-Wallis test revealed statistically significant differences between the mean responses of the sample on axes 2 and 3 based on the Marital Status of the Parent at the 0.05 significance level *H*(2) = 8.26, *P* = 0.02; *H*(2) = 6.35, *P* = 0.04, respectively. Thus, we reject the null hypothesis.

**Table 4:** Kruskal–Wallis test, the significance of the differences in the mean of sample`s responses according to Parent`s Marital status.

Questionnaire axes	Groups	*n*	Mean Rank.	*H*
				*Df*
				*P*
Axis2.The family has a significant role in promoting an active lifestyle (i.e., exercise, physical and sports activities) among their children	Married	343	208.43	8.26*
	Divorced	11	113.73	2
	Widower	51	185.73	0.02
Axis3. Parental awareness of community resources needed to promote active lifestyles in children	Married	343	205.40	6.35*
	Divorced	11	115.36	2
	Widower	51	205.77	0.04
Axis4. The effectiveness of community activities and awareness programs to promote a children`s active lifestyle	Married	343	204	2.65
	Divorced	11	146.73	2
	Widower	51	206.77	0.27

* Significant at the 0.05 level.

#### The significance of the differences in the mean of the sample’s responses among each pair group according to the parent’s marital status

From Table 5, the Mann-Whitney test indicated that the family played a more significant role in promoting an active lifestyle (i.e., exercise, physical and sports activities) among their children for the Married Parent category (*Mdn* = 2.87) than for the Divorced Parent category (*Mdn* = 2.47) *U* = 984, *P* = 0.01. Moreover, the Mann-Whitney test indicated that families recognize that community resources are needed to promote children’s active lifestyles more often in the Married Parent category (*Mdn* = 3.08) than in the Divorced Parent category (*Mdn* = 2.58) *U* = 1052, *P* = 0.01.

**Table 5:** The Mann-Whitney test to determine the significant difference in the mean of sample`s responses among each pair group according to Parent`s Marital status.

Questionnaire axes	The Mann-Whitney *U*, *P-Value*		
	Groups	Married	Divorced
Axis2.The family has a significant role in promoting an active lifestyle (i.e., exercise, physical and sports activities) among their children	Divorced	984*	
		0.01	
	Widower	7786	201
		0.20	0.14
Axis3. Parental awareness of community resources needed to promote active lifestyles in children	Divorced	1052*	
		0.01	
	Widower	8734.50	151
		0.99	0.17

* Significant at the 0.05 level

#### The significance of the differences in the mean of the sample’s responses according to parent’s education level

*H*_1_: There are statistically significant differences between the mean responses of the sample on axes 2 and 3 based on the Parent’s Education level at the 0.05 significance level.

From Table 6, The Kruskal-Wallis test revealed that there are statistically significant differences between the mean responses of the sample on axis 2 based on the father’s and mother’s education level at the 0.05 significance level *H*(3) = 15.71, *p≤0*.*01*; *H*(3) = 9.23, *P* = 0.03, respectively. In addition, the Kruskal-Wallis test indicated significant differences between the mean responses of the sample on axis 4 based on the father’s Education level *H*(3) = 8.31, *P* = 0.04. Thus, we reject the null hypothesis.

**Table 6:** Kruskal–Wallis test, the significance of the differences in the mean of sample`s responses according to Parent`s education level.

Questionnaire axes	Groups	Father’s education level			Mother’s education level		
		*n*	Mean Rank.	*H* *df* *P*	*N*	Mean Rank.	*H* *df* *P*
Axis2.	Postgraduate	55	213.38	15.71* 3 0.00	29	255.12	9.23* 3 0.03
	University graduate	237	215.99		239	205.20	
	Secondary education	79	185.77		64	199.67	
	Intermediate education	34	137.34		73	178.01	
Axis3.	Postgraduate	55	185.15	3.10 3 0.38	29	163.10	5.49 3 0.14
	University graduate	237	209.75		239	205.67	
	Secondary education	79	203.92		64	221.30	
	Intermediate education	34	182.68		73	194.08	
Axis4.	Postgraduate	55	188.95	8.31* 3 0.04	29	189.66	0.84 3 0.84
	University graduate	237	194.66		239	201.11	
	Secondary education	79	235.85		64	207.62	
	Intermediate education	34	207.56		73	210.45	

* Significant at the 0.05 level.

#### The significant difference in the mean of the sample’s responses among each pair group according to the parent’s education level

From Table 7, The Mann-Whitney test revealed significant differences in responses on the general average of axis 2 entitled “The family has a significant role in promoting an active lifestyle among their children,” for father’s university graduate (*Mdn* = 2.93) versus secondary education (*Mdn* = 2.73) *U* = 7925.50, *P* = 0.04.

**Table 7:** The Mann-Whitney test to determine the significant difference in the mean of sample`s responses among each pair group according to Parent`s education level.

Questionnaire axes	The Mann-Whitney *U*, *P-Value*			
	Father’s education level			
	Groups	Postgraduate	University graduate	Secondary education
Axis2.The family has a significant role in promoting an active lifestyle among their children	University graduate	6434		
		0.88		
	Secondary education	1905.50	7925.50*	
		0.23	0.04	
	Intermediate education	602.50*	2471*	1001*
		0.01	0.00	0.03
Axis4. The effectiveness of community activities and awareness programs to promote a children`s active lifestyle	University graduate	6361.50		
		0.78		
	Secondary education	1657*	7468.50*	
		0.02	0.01	
	Intermediate education	833.50	3789	1156.50
		0.39	0.57	0.24
	Mother’s education level			
	Groups	Postgraduate	University graduate	Secondary education
Axis2.The family has a significant role in promoting an active lifestyle among their children	University graduate	2612*		
		0.03		
	Secondary education	672*	7429.50	
		0.03	0.72	
	Intermediate education	656*	7563	2074.50
		≤0.01	0.08	0.26

* Significant at the 0.05 level.

It is also clear that the lowest group responding to the axis 2 is the father’s intermediate education group (*Mdn* = 2.57), while the highest group is University graduates (*Mdn*). = 2.93). It was followed by the Postgraduate group (*Mdn* = 2.87), then the Secondary education group (*Mdn* = 2.73), where there were statistically significant differences at the significance level of 0.05, and the level of significance of the differences between them was *U* = 2471, *p≤0*.*01*; *U* = 602.50, *P* = 0.01; *U* = 1001, *P* = 0.03, respectively.

As the table shows, there are significant differences in responses about axis 4 entitled “the effectiveness of community activities and awareness programs to promote children’s active lifestyle” for Father’s Secondary education (*Mdn* = 3.85) as compared to Postgraduate (*Mdn* = 3.43), *U* = 1657, *P* = 0.02. Also, more than for University graduates (*Mdn* = 3.43), *U* = 7468.50, *P* = 0.01.

For the mother’s education level, it is clear that the lowest group responding on axis 2 is the mother’s intermediate education group (*Mdn* = 2.73), while the highest group is postgraduate (*Mdn*. = 3.00). It was followed by the Secondary education group (*Mdn* = 2.97), then the university graduate group (*Mdn* = 2.87), where there were statistically significant differences at the significance level of 0.05; the level of significance of the differences between them was *U* = 656, *p≤0*.*01*; *U* = 672, *P* = 0.03; *U* = 2612, *P* = 0.03, respectively.

## Discussion

The lack of interest among families in Qassim Province in children’s regular daily physical activity was noteworthy. The children typically practiced physical activity two to three times a week for less than 1 h [6, 7]. These results are consistent with those of Al-Hazzaa and Al-Rasheedi [29] and Al-Kadi et al. [30]. Other studies [31, 32] have noted an alarmingly high prevalence of overweight and obesity among Saudi children and adolescents.

We believe that awareness programs promoting children’s regular physical activity have not achieved the desired goals, consistent with Al-Hazzaa and AlMarzooqi [33]. These initiatives have been fragmented, short-lived, and lack a coordinating body. Most physical activity initiatives also lack objective evaluations of their outcomes.

In Qassim Province, most children practice sports and physical activity outside the home with their peers, and 4% of children practice sports in government clubs. In addition, 3.7% are members of neighborhood clubs. These results are consistent with the study of Al Harthi and El-Araby [34] and Spaaij et al. [35].

Official clubs have other benefits beyond physical activity. They help build a child’s personality, develop values of belonging, transfer knowledge and behaviors, and refine life skills and social communication skills [36–38]. Also, sports coaches play an important role in treating children’s postural deformities [35, 39, 40], psychological problems, and behavior [39, 40]. Moreover, these clubs provide safety and security [41–43].

There is a need to increase families’ awareness of the benefits of registering their children in official sports federations. This supports the child’s self-realization and sporting ambition, enhancing self-esteem [44–46].

We found that most families provide motor games and appropriate sports equipment for the child at home, providing opportunities for free play that enhances movement, physical activity, and social and cognitive values [47]. The results also show the availability of appropriate sports equipment for the parents at home and that the parents practice sports and physical activity, though irregularly. Arlinghaus and Johnston [48] emphasize that parents’ habits are an important component of changing children’s lifestyle behavior. Rodrigues et al. [49] found that the activity of parents was significantly associated with more frequent participation in a greater number of sports in sons. Thus, the passive lifestyle practiced by the parents is transmitted to the children as a permanent habit and becomes difficult to change [50–53].

Consistent with the literature [54–57], the families in our study were aware of the importance of encouraging children to engage in sports activities, healthy diets and sleep habits, and social and psychological stability. Consistent with other studies [58, 59], the parents were aware of community resources to promote active lifestyles in children and the effectiveness of the school in encouraging children to exercise and engage in physical activity. The parents’ agreed that neighborhood clubs and the availability of public gardens provide children with opportunities to practice various sports activities, consistent with Al-Silwi [60] and others [61–64].

Coombes et al. [65] report living near green spaces (formal gardens) encourages physical activity and reduces the rates of overweight or obesity and that access to green spaces in urban areas may help enhance the residents’ physical activity. Thus, policies assisting with providing devices and tools in most public parks may further promote active lifestyles.

We found that families lack awareness of the opportunities offered by government clubs for children to practice sports and physical activity. Thus, there seems to be a lack of adequate information regarding the role of sports clubs in promoting an active lifestyle for children.

The parents’ expressed a desire to employ crews that enhance children’s movement and recreational activities in shopping centers. This was confirmed by the study of Pospěch [66]. White [67] points out the need to provide walking spaces and places with simulators for various sports, urging shopping mall-goers to show their skills and challenge their abilities. Park et al. [68] found that green parks independently enhance physical activity and positively influence healthy lifestyles. Therefore, public-private partnerships for physical activity and health in urban planning must be emphasized.

The parents in our study were keen to obtain the necessary information about children’s active lifestyles. This finding is encouraging given that of Al-Hazzaa and AlMarzooqi [33] conclude that more intensive efforts and initiatives are needed to enhance physical activity and reduce sedentary behaviors among the Saudi population. Such efforts could counter the spread of physical inactivity and the lack of free time to engage in physical activities among Saudis [69].

Our results (fourth question) indicate that the family plays an important role in promoting an active lifestyle among children. Our findings are consistent with those of McVeigh et al. [70] and Vereecken et al. [71]; double-parent homes and a higher level of education of parents are associated with healthy lifestyles.

## Conclusions

This questionnaire study reveals a concerning lack of interest and awareness among parents about appropriate levels of physical activity for their children. Nevertheless, most parents report being keen to learn more. Further efforts are urgently needed to reverse a growing tendency towards inactivity and unhealthy lifestyles among Saudis.

## Limitations

Due to the COVID-19 pandemic, we have had to rely only on the responses of parents through an online survey in place of other research tools, such as interviews and observations of children.

## Implications

To promote an active lifestyle for children, there is a need to determine why some families are hesitant to enroll their children in sports clubs. Additionally, there is a need to develop media and awareness campaigns for families to achieve the desired goals of developing an active lifestyle for children, which are approved by the WHO and Saudi Ministry of Health standards and are consistent with families’ low educational levels.

## Supporting information

S1 Table Description of the study sample. (PDF)

S2 Table Five-point Likert rating scale interpretation of weighted mean scale. (PDF)

S3 Table Pearson bivariate correlation between every item and their axis (n = 30). (PDF)

S4 Table Cronbach’s Alpha values to determine the questionnaire reliability (n = 30). (PDF)

S5 Table One sample Kolmogorov-Smirnov normality test. (PDF)
