# Supplementary material for: Developing an active lifestyle for children considering the Saudi vision 2030: The family’s point of view
Source: PLoS One. 2022 Sep 26;17(9):e0275109. doi: 10.1371/journal.pone.0275109 (PMC9512172; doi:10.1371/journal.pone.0275109)
Supplement: S1 Table — (PDF) [file pone.0275109.s001.pdf]

S1 Table. Description of the study sample.

| Gender | Frequencies | % |
|---|---|---|
| Girl | 113 | 27.9 |
| Boy | 292 | 72.1 |
| Total | 405 | 100.0 |
| Age [1] | Frequencies | % |
| Early childhood (Ages 3-5) | 62 | 15.3 |
| Middle childhood (Ages 6-8) | 122 | 30.1 |
| Late childhood (Ages 9-11) | 221 | 54.6 |
| Total | 405 | 100.0 |
| Type of school | Frequencies | % |
| Public school | 309 | 76.3 |
| Private school | 26 | 6.4 |
| International school | 13 | 3.2 |
| Disabled children’s School | 1 | 0.2 |
| Not enrolled | 56 | 13.8 |
| Total | 405 | 100.0 |
| Health status | Frequencies | % |
| Normal | 394 | 97.28 |
| Suffers from a chronic disease | 7 | 1.7 |
| Disabled children’s | 4 | 1.0 |
| Total | 405 | 100.0 |
| Father’s education level | Frequencies | % |
| Postgraduate | 55 | 13.6 |
| University graduate | 237 | 58.5 |
| Secondary education | 79 | 19.5 |
| Intermediate education | 34 | 8.4 |
| Total | 405 | 100.0 |
| Mother’s education level | Frequencies | % |
| Postgraduate | 29 | 7.2 |
| University graduate | 239 | 59.0 |
| Secondary education | 64 | 15.8 |
| Intermediate education | 73 | 18.0 |

| | | |
|---|---|---|
| Total | 405 | 100.0 |
| Father’s occupation | Frequencies | % |
| Governmental job | 243 | 60.0 |
| Private job | 94 | 23.2 |
| Military job | 68 | 16.8 |
| Total | 405 | 100.0 |
| Mother’s occupation | Frequencies | % |
| Governmental job | 175 | 43.2 |
| Private job | 21 | 5.2 |
| Housewife | 209 | 51.6 |
| Total | 405 | 100.0 |
| Marital Status of Parent | Frequencies | % |
| Married | 343 | 84.7 |
| Widower | 51 | 12.6 |
| Divorced | 11 | 2.7 |
| Total | 405 | 100.0 |
| The number of children from (3 to 12) years in the family | Frequencies | % |
| One Child | 134 | 33.1 |
| Two children | 156 | 38.5 |
| Three children | 58 | 14.3 |
| Four children or more | 57 | 14.1 |
| Total | 405 | 100.0 |

1. Armstrong T. The Human Odyssey: Navigating the Twelve Stages of Life: Dover Publications; 2019.
