# Supplementary material for: Developing an active lifestyle for children considering the Saudi vision 2030: The family’s point of view
Source: PLoS One. 2022 Sep 26;17(9):e0275109. doi: 10.1371/journal.pone.0275109 (PMC9512172; doi:10.1371/journal.pone.0275109)
Supplement: S2 Table — (PDF) [file pone.0275109.s002.pdf]

S2 Table. Five-point Likert Rating Scale Interpretation of Weighted Mean Scale

	Range-Value	Verbal Interpretation
5	4.50-5.00	Strongly Agree
4	3.50-4.49	Agree
3	2.50-3.49	Neutrally
2	1.50-2.49	Disagree
1	1.00-1.49	Strongly Disagree
