# Supplementary material for: Developing an active lifestyle for children considering the Saudi vision 2030: The family’s point of view
Source: PLoS One. 2022 Sep 26;17(9):e0275109. doi: 10.1371/journal.pone.0275109 (PMC9512172; doi:10.1371/journal.pone.0275109)
Supplement: S3 Table — (PDF) [file pone.0275109.s003.pdf]

S3 Table. Pearson bivariate correlation between every item and their axis (n = 30).

**Variable**	***M*** ***SD***	***R***	***p***
Axis 2. The family has a significant role in promoting an active lifestyle (i.e., exercise, physical and sports activities) among their children			
1- Is the Child registered in one of the sports federations?	1.03 0.18	0.62**	≤0.01
2- Does your Child engage in sports or any physical activity?	1.87 0.73	0.51**	≤0.01
3- How often do the child exercise sports and physical activity per week?	2.00 0.74	0.58**	≤0.01
4- How long do the child exercise sports and physical activity per day?	2.00 0.79	0.55**	≤0.01
5- Where the Child engages in sports and physical activity?	2.00 0.69	0.57**	≤0.01
6- Is there kinetic toys and sports equipment suitable for the Child at home?	1.17 0.38	0.65**	≤0.01
7- Is there sports equipment suitable for parents at home?	1.10 0.30	0.71**	≤0.01
8- Are parents keen on exercise, sports, and physical activity?	1.27 0.58	0.57**	≤0.01
9- Does the family share and encourage the child to practice kinetic games at home?	3.83 0.91	0.43*	≤0.01
10- Does the family set a specific time for the child to practice sedentary activities (watching TV - PlayStation - Smartphones, etc.)?	4.07 1.11	0.41*	≤0.01
11- Is the family aware of sufficient information about the healthy food system for children?	3.90 1.03	0.51**	≤0.01
12- Does the family ensure that the child follows a healthy diet?	4.00 0.91	0.63**	≤0.01
13- Is The family aware of the necessary information about the child's healthy sleep habits?	4.03 0.89	0.61**	≤0.01

14- Is the family keen the child's healthy sleep habits (sleep early - sleep enough)?	3.80<br>1.03	0.44^{*}	≤0.01
15- Do you think that parents' social and psychological stability enhances the children's active life?	4.30<br>0.60	0.43^{*}	≤0.01
Axis 3. Parental awareness of community resources needed to promote active lifestyles in children			
1- Governmental sports clubs are influential and provide children with opportunities to practice various sports activities	3.40<br>1.22	0.73^{**}	≤0.01
2- The District Sports Club project is influential and provides children with opportunities to practice various sports activities	3.73<br>1.20	0.67^{**}	≤0.01
3- Fees for the child's participation in government sports clubs appropriate to the family's income level.	3.50<br>1.04	0.61^{**}	≤0.01
4- Fees for the child's participation in the District Sports Clubs appropriate to the family's income level.	3.73<br>0.94	0.62^{**}	≤0.01
5- Fees for the child's participation in private sports clubs and academies commensurate with the family's income level.	3.03<br>1.16	0.76^{**}	≤0.01
6- Government sports clubs have specialized qualified trainers to train children in various sports activities (football - basketball - volleyball - handball - swimming - field and track competitions - gymnastics - etc.)	3.30<br>1.09	0.62^{**}	≤0.01
7- The District Sports Clubs have specialized qualified trainers to train children in various sports activities (football - basketball - volleyball - handball - swimming - field and track competitions - gymnastics.)	3.23<br>1.04	0.70^{**}	≤0.01
8- The school encourages children to exercise and physical activity and rehabilitating them physically and healthily through physical education and sports activities.	3.73<br>1.11	0.79^{**}	≤0.01
9- In every residential neighborhood, a public park enables the family to practice physical activity.	3.77<br>1.16	0.47^{**}	≤0.01
10- In Public Parks, There is sports equipment suitable for children to practice physical activity.	3.53<br>1.11	0.82^{**}	≤0.01

11- In public parks, Guide instructional panels are available for the use of sports equipment, and there are safety and security factors for children to practice physical activity.	3.23<br>1.33	0.79**	≤0.01
12. Private sports clubs and academies are available enough for children to exercise in various sports activities, and it is near the family home.	3.07<br>1.26	0.80**	≤0.01
Axis 4. The effectiveness of community activities and awareness programs to promote a children`s active lifestyle			
1- Does community activities such as (Dates Festival - Buraidah spring, etc.) organized by responsible government agencies allow the child to participate in physical activities and sports competitions that promote an active lifestyle?	3.47<br>1.17	0.74**	≤0.01
2 - It is necessary to employ an animation crew in public parks to organize sports programs, workshops, and creative activities for visitors to enhance the active lifestyle of children	3.23<br>1.30	0.91**	≤0.01
3- It is necessary to employ an animation crew in Shopping malls to organize sports programs and activities for visitors to enhance the active lifestyle of children.	3.43<br>1.07	0.79**	≤0.01
4- The specialized organizations (Sports Ministry - Health Ministry, etc.) provide the necessary awareness programs to help the family in promoting an active lifestyle for children	3.93<br>1.05	0.56**	≤0.01
5- Are you keen to follow up awareness programs through various media to develop the necessary information about children's active lifestyles?	3.87<br>1.17	0.80**	≤0.01
6- Is the family has sufficient information to meet the World Health Organization and the Saudi Ministry of Health standards regarding the child's practice of physical activity?	3.53<br>1.01	0.89**	≤0.01
7- Is the family committed to achieving the World Health Organization and the Saudi Ministry of Health standards regarding the child's practice of physical activity?	3.00<br>1.02	0.79**	≤0.01

** Correlation is significant at the 0.01 level (2-tailed).

* Correlation is significant at the 0.05 level (2-tailed).
