# Supplementary material for: Developing an active lifestyle for children considering the Saudi vision 2030: The family’s point of view
Source: PLoS One. 2022 Sep 26;17(9):e0275109. doi: 10.1371/journal.pone.0275109 (PMC9512172; doi:10.1371/journal.pone.0275109)
Supplement: S4 Table — (PDF) [file pone.0275109.s004.pdf]

S4 Table. Cronbach's Alpha values to determine the questionnaire reliability (n = 30).

**Variable**	**Cronbach's Alpha**	**Cronbach's Alpha Based on Standardized Items**
Axis 2. The family has a significant role in promoting an active lifestyle (i.e., exercise, physical and sports activities) among their children		0.807
Axis 3. Parental awareness of community resources needed to promote active lifestyles in children	0.901	
Axis 4. The effectiveness of community activities and awareness programs to promote a children`s active lifestyle	0.821	
