# Supplementary material for: Developing an active lifestyle for children considering the Saudi vision 2030: The family’s point of view
Source: PLoS One. 2022 Sep 26;17(9):e0275109. doi: 10.1371/journal.pone.0275109 (PMC9512172; doi:10.1371/journal.pone.0275109)
Supplement: S5 Table — (PDF) [file pone.0275109.s005.pdf]

S5 Table. One sample Kolmogorov-Smirnov Normality test

Questionnaire axes	Statistic	*df*	Sig.
Axis2.The family has a significant role in promoting an active lifestyle (i.e., exercise, physical and sports activities) among their children	.053	405	.01
Axis3. Parental awareness of community resources needed to promote active lifestyles in children	.071	405	≤0.01
Axis4. The effectiveness of community activities and awareness programs to promote a children`s active lifestyle	.066	405	≤0.01
